# Use of patient-reported outcome measures (PROMs) by orthopedic surgeons in Saudi Arabia

**DOI:** 10.1186/s13018-020-02135-1

**Published:** 2020-12-10

**Authors:** Fayez Alshehri, Abdulaziz Alarabi, Mohammed Alharthi, Thamer Alanazi, Ahmed Alohali, Mohammad Alsaleem

**Affiliations:** 1 College of Medicine, Imam Muhammad ibn Saud Islamic University, Riyadh, Kingdom of Saudi Arabia; 2 Department of Orthopedic Surgery, King Fahad Hospital, Hofuf, Kingdom of Saudi Arabia

**Keywords:** PROM, Total joint arthroplasty, TJA, Patient-reported outcome measures, Use of PROMs, Use by orthopedic surgeons, Saudi Arabia

## Abstract

**Background:**

There is increasing literature on the usefulness of patient-reported outcome measures (PROMs), but far fewer studies to determine their use by orthopedic surgeons and the barriers they face in applying PROMs in their daily clinical activity.

**Methods:**

Cross-sectional study using a questionnaire that was distributed in both soft and hard copy formats to a sample of 262 orthopedic surgeons. Participants included orthopedic surgeons who are employed by the Ministry of Health (MOH) in Riyadh and the Eastern Province, Saudi Arabia. The questionnaire was distributed through on-site visitations to orthopedic departments in MOH hospitals as well as through online correspondence by email, WhatsApp, and social media.

**Results:**

The study sample included 262 orthopedic surgeons (13.7% females and 86.3% males). Surgeons aged < 34, 35–44, and 45–54 years old represented 28.66%, 38.9%, and 20.2% of the study sample, respectively. The majority of the included surgeons did not use PROMs (69.1%), and some (17.2%) used it for research purposes. Only 5% used it regularly in daily clinical work.

**Conclusion:**

The clinical use of PROMs among orthopedic surgeons was negligible, even though an overwhelming majority were interested in using PROMs. The reasons provided included a lack of knowledge on how to use PROMs and the perception that it is too time-consuming to add to regular clinical routine. There should be more efforts towards training surgeons on how to use PROMs, whereas increasing compatibility with existing software tools used by MOH hospitals may help offset time-related reservations.

## Background

As the volume of total joint arthroplasty (TJA) procedures performed in countries across the world continues to increase [1], so has the demand for further evidence of when they are necessary [2]. Patient-reported outcome measures (PROMs) are tools that enable patients to self-report their functional status, pain, and other valuable domains related to their quality of life [3]. With the possibility to be used in both pre- and postoperative settings, PROMs can assist in determining patient satisfaction after TJA by documenting the changes in score and serve as an indicator of the surgical intervention’s efficacy [4].

While PROMs are not without their challenges, they remain the best objective tool available for measuring patient centered outcomes according to the International Society of Arthroplasty Registries and various clinicians [5, 6]. Some of these challenges include choosing the right PROMs that fit the patient population, lack of standardization, and their readability [7, 8].

Still, PROMs continue to empower patients by giving them the ability to become more involved than ever when it comes to their health, by directly contributing to medical and surgical assessments [9].

There is substantial literature surrounding PROM use with regard to evaluating the outcomes in arthroplasty [10–13], but few studies are available regarding its use by orthopedic surgeons for clinical purposes and the barriers they face. Approximately 16% of surgeons of various specialties in the Middle East currently use PROMs [14], whereas as little as 5.9% of orthopedic and neurosurgeons routinely use PROMs in the Middle East [15].

In both studies, the Middle East had the lowest percentage of surgeons who use PROMs, with North America and Europe being the highest. Both studies had surgeons citing the lack of time and structural constraints in their institutions as the biggest barriers against using PROMs. The contrast between the similarity in reported barriers and the variation in PROM use across different regions demonstrates that while surgeons ultimately face similar obstacles, the root cause may vary. This creates a need to specifically examine each country separately in order to discern the unique causes that lead to the previously reported barriers. This is especially necessary in regions with variable levels of healthcare such as the Middle East [16].

There are no studies regarding PROM use by orthopedic surgeons in Saudi Arabia. The aim of this study is to identify the prevalence of PROM use by orthopedic surgeons in Saudi Arabia, the reasons behind the use or lack thereof, and the barriers they may face.

## Methods

### Design

Using a cross-sectional research design with a convenience sampling technique, this study examined the use of patient-reported outcome measures (PROMs) by orthopedic surgeons with regard to its implementation into regular clinical activity and/or using PROMs for research purposes, in addition to barriers they may face, as well as their perceptions regarding PROMs.

A quantitative research approach was conducted using a self-administered survey that is available in both soft and hard copy formats via Google Forms. A prevalidated questionnaire was adapted from a previous study [14]. After obtaining permission to use the questionnaire, it was slightly modified for more applicability, by removing a question regarding mandatory use of PROMs since no such regulations exist by the Ministry of Health (MOH), and a question on whether PROMs are substitute measurements of clinical outcomes. No pilot study was conducted after the removal of these two questions.

### Study questionnaire

The survey included thirty-two items, beginning with a cover letter that provided the details on the objectives of the study, assurances regarding the confidentiality, and anonymity of the collected data. Informed consent was required and collected before moving on to the survey. Participants then proceeded to questions of the first section starting with socio-demographic information, as well as asking orthopedic surgeons on their familiarity with existing universal PROMs and specific PROMs for trauma and orthopedic patients. The last part of the first section asked whether surgeons use PROMs or not, the frequency of the use, and the purposes behind the use (whether it is for clinical activity or research purposes). Branching logic was used to move respondents to section 2-A (surgeons who used PROMs in clinical routine) and section 2-B (surgeons who did not use PROMs in clinical routine) based on their answers in the first section. Both sections contained statements of agreement on reasons for their use of PROMs or lack thereof. In addition, information on two further aspects of PROMs was questioned in the third and fourth sections: aspects that are important in order to implement PROMs in daily clinical routine and the reasons why PROMs are not used more often in daily clinical routine. The questionnaire ended with a question that asked whether surgeons would be interested in using PROMs in daily clinical routine if there was a tool that could overcome the barriers they reported.

Data collection took place between 28 May 2019 and 30 November 2019. The distribution of the questionnaire entailed both on-site visitations to orthopedic departments of hospitals, as well as online correspondence through email, WhatsApp, and social media.

### Statistical analysis and sample size calculation

The margin of error at 95% confidence (expressing the amount of random sampling error) was computed. Percentages for all categorical variables were computed. To examine the associations between two categorical variables, the chi-square test of independence was used to evaluate the association of “gender,” “age,” “years of clinical experience,” and “area of work” with (A) the familiarity with existing universal PROMs, (B) the familiarity with specific PROMs for trauma and orthopedic patients, and (C) the current use of PROMs in clinical work. The significance level was set at *p* < 0.05. All statistical analyses were performed using R v 3.6.2.

The target population of the study was all the orthopedic surgeons who are employed by MOH hospitals in Riyadh and the Eastern Province. The total number of registered orthopedic surgeons who are employed by the MOH in Riyadh and the Eastern Province was 399 according to the MOH statistical yearbook during the time of this study. Sample size calculation was performed using Epi-INFO (Benichou, 2014). Based on an expected prevalence of 50% (expected prevalence for the use of PROMs) and a significance level of 0.05, we hypothesized that a sample size of 240 is needed to assess the current use of PROMs with an acceptable error of 4%. We recruited 262 orthopedic surgeons in the current analysis which fulfilled the pre-calculated estimate.

## Results

The study sample included 262 orthopedic surgeons (13.7% females and 86.3% males). Surgeons aged < 34, 35–44, and 45–54 years old represented 28.66%, 38.9%, and 20.2% of the study sample, respectively. Surgeons from Riyadh represented 63.7% of the study sample. Less than half of the included surgeons were aware of the existing universal PROMs (40.8%) and slightly more than half (58%) were familiar with specific PROMs for trauma and orthopedic patients. The majority of the included surgeons did not use PROMs (69.1%), and some (17.2%) used it for research purposes. Only 5% used it regularly in daily clinical work (Table 1).

**Table 1:** Summary of demographic and working experience of the study sample

	***N*** (%)
**Gender**	
Female	36 (13.7%)
Male	226 (86.3%)
**Age (years)**	
< 34	75 (28.66%)
35 to 44	102 (38.9%)
45 to 54	53 (20.2%)
> 55	32 (12.21%)
**Years of clinical experience (years)**	
0 to 4	44 (16.8%)
5 to 9	57 (21.8%)
10 to 14	69 (26.3%)
15 to 19	40 (15.3%)
20 or more	52 (19.8%)
**Familiarity with existing universal PROMs**	
No	155 (59.2%)
Yes	107 (40.8%)
**Familiarity with specific PROMs for trauma and orthopedic patients**	
No	110 (42.0%)
Yes	152 (58.0%)
**Current use of PROMs**	
Both, in daily clinical work and research	6 (2.29%)
No, I do not use PROMs	181 (69.1%)
Yes, for research purposes only	45 (17.2%)
Yes, infrequently in daily clinical work	17 (6.49%)
Yes, regularly in daily clinical work	13 (4.96%)

Univariate analysis showed that age was significantly associated with knowledge regarding universal (*P* = 0.002) and disease -specific (*P* = 0.031) PROMs. The results showed a consistent increase in the knowledge regarding the universal PROMs. For disease-specific PROMs, knowledge was higher in all age groups compared to the < 34 years age group. Area of work did not show a statistically significant association with knowledge regarding disease specific PROMs (*P* = 0.054). Similarly, years of experience did not show a statistically significant association with knowledge regarding the universal PROMs (*P* = 0.058) (Table 2).

**Table 2:** Factors associated with familiarity and current use of PROMs

	Familiarity with universal PROM			Familiarity with specific PROMs			Current use		
	No	Yes	***P***	No	Yes	***P***	No	Yes	***P***
	***N = 155***	***N = 107***		***N = 110***	***N = 152***		***N = 226***	***N = 36***	
**Gender**			0.242			0.386			0.436
Female	25 (69.4%)	11 (30.6%)		18 (50.0%)	18 (50.0%)		33 (91.7%)	3 (8.33%)	
Male	130 (57.5%)	96 (42.5%)		92 (40.7%)	134 (59.3%)		193 (85.4%)	33 (14.6%)	
**Age (years)**			**0.002**			**0.031**			**0.025**
< 34	55 (73.3%)	20 (26.7%)		41 (54.7%)	34 (45.3%)		70 (93.3%)	5 (6.67%)	
35 to 44	58 (56.9%)	44 (43.1%)		37 (36.3%)	65 (63.7%)		90 (88.2%)	12 (11.8%)	
45 to 54	31 (58.5%)	22 (41.5%)		23 (43.4%)	30 (56.6%)		42 (79.2%)	11 (20.8%)	
> 55	11 (34.4%)	21 (65.6%)		9 (28.1%)	23 (71.9%)		24 (75.0%)	8 (25.0%)	
**Clinical experience (years)**			0.058			0.162			0.137
0 to 4	31 (70.5%)	13 (29.5%)		26 (59.1%)	18 (40.9%)		39 (88.6%)	5 (11.4%)	
5 to 9	39 (68.4%)	18 (31.6%)		23 (40.4%)	34 (59.6%)		52 (91.2%)	5 (8.77%)	
10 to 14	34 (49.3%)	35 (50.7%)		27 (39.1%)	42 (60.9%)		62 (89.9%)	7 (10.1%)	
15 to 19	25 (62.5%)	15 (37.5%)		15 (37.5%)	25 (62.5%)		30 (75.0%)	10 (25.0%)	
20 or more	26 (50.0%)	26 (50.0%)		19 (36.5%)	33 (63.5%)		43 (82.7%)	9 (17.3%)	
**Area of work**			0.656			0.054			1.000
Eastern Province	54 (56.8%)	41 (43.2%)		32 (33.7%)	63 (66.3%)		82 (86.3%)	13 (13.7%)	
Riyadh	101 (60.5%)	66 (39.5%)		78 (46.7%)	89 (53.3%)		144 (86.2%)	23 (13.8%)	

Statistical analysis was performed using the chi-square test of independence

Current use was defined as using PROMs infrequently/regularly/in clinical routine and research

Only 36 surgeons used PROMs in their clinical routine (irregularly or regularly or both in daily clinical work and research). Their most perceived benefits of using PROMs were prioritizing clinical problems (97% agreed or strongly agreed) and facilitating communication between surgeons and patients (94% agreed or strongly agreed). The least perceived advantage was identifying patients’ preferences (72% agreed or strongly agreed). The percentage of agreement for the remaining five statements ranged from 81 to 89% (Table 3).

**Table 3:** Detailed reasons for orthopedic surgeons who collect PROMs in clinical routine

	SD	D	N	A	SA
PROMs can help to prioritize clinical problems.	0 (0%)	0 (0%)	1 (2.78%)	24 (66.7%)	11 (30.6%)
PROMs can help to facilitate communication between the doctor and the patient.	0 (0%)	1 (2.78%)	1 (2.78%)	21 (58.3%)	13 (36.1%)
PROMs can screen for potential patient problems.	0 (0%)	0 (0%)	4 (11.1%)	20 (55.6%)	12 (33.3%)
PROMs can identify a patient’s preferences.	0 (0%)	0 (0%)	10 (27.8%)	17 (47.2%)	9 (25.0%)
PROMs monitor changes/responses to treatment.	0 (0%)	2 (5.56%)	5 (13.9%)	18 (50.0%)	11 (30.6%)
PROMs monitor the general healthcare status of my patients and their healthcare status changes.	0 (0%)	1 (2.78%)	5 (13.9%)	22 (61.1%)	8 (22.2%)
PROMs help to monitor the quality of healthcare provision.	0 (0%)	1 (2.78%)	4 (11.1%)	21 (58.3%)	10 (27.8%)
PROMs are useful for national/international comparison and benchmarking.	0 (0%)	2 (5.56%)	3 (8.33%)	21 (58.3%)	10 (27.8%)

*SD* strongly disagree, *D* disagree, *N* neutral, *A* agree, *SA* strongly agree

The top two perceived barriers against using PROMs in daily clinical routine were the long time needed to fill out PROMs (48.9% agreed and 14.1% strongly agreed) and the significant change needed to implement PROMs (49.6% agreed and 12.2% strongly agreed) (Table 4).

**Table 4:** Perceived barriers for not using PROMs in daily clinical routine

	SD	D	N	A	SA
Data from PROMs are subjective; they cannot adequately reflect an individual’s situation.	10 (3.82%)	71 (27.1%)	88 (33.6%)	74 (28.2%)	19 (7.25%)
Orthopedic surgeons lack the necessary skills to interpret and use the information given by these instruments.	18 (6.87%)	73 (27.9%)	69 (26.3%)	86 (32.8%)	16 (6.11%)
To fill out PROMs is time-consuming and burdensome for the patients.	3 (1.15%)	31 (11.8%)	63 (24.0%)	128 (48.9%)	37 (14.1%)
Implementing PROMs would require significant changes in the structure of the basic clinical routine of healthcare providers as well as being costly.	2 (0.76%)	41 (15.6%)	57 (21.8%)	130 (49.6%)	32 (12.2%)

*SD* strongly disagree, *D* disagree, *N* neutral, *A* agree, *SA* strongly agree

**Fig. 1:**
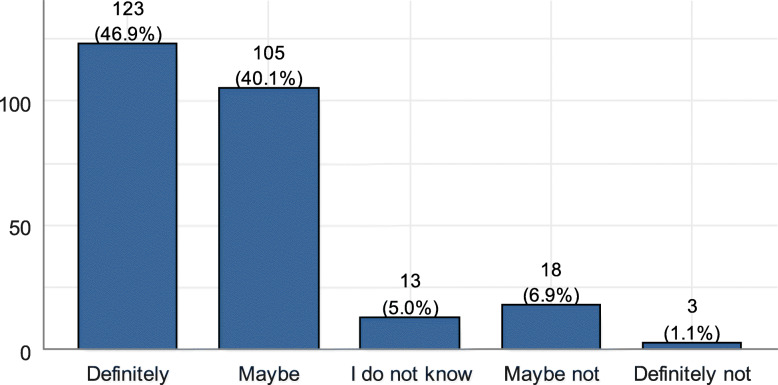
Interest in using PROMs in the presence of a tool that can overcome barriers

The results showed that 87% of the included surgeons would be interested in using PROMs if there was a tool that can overcome the barriers to its use (Fig. 1).

## Discussion

PROM use by surgeons has already been documented in several countries and regions [14, 15, 17], yet no such data exists for Saudi Arabia. Understanding the current status of PROMs among orthopedic surgeons in Saudi Arabia, its use or lack thereof, and the underlaying rationale can lay the groundwork for future research. A sample of 262 orthopedic surgeon who work in Saudi Arabia’s Ministry of Health (MOH) hospitals in Riyadh and the Eastern Province revealed that familiarity with specific PROMs for trauma and orthopedic is higher (58%) than familiarity with universal PROMs (40.8%), yet a majority (86.3%) did not use PROMs in their clinical routine.

Our study showed that orthopedic surgeons in the Eastern Province had a higher familiarity with both universal (43.2%) and disease-specific (66.3%) PROMs compared to Riyadh’s orthopedic surgeons. Despite this gap in familiarity, it appears that there was no difference in the current use of PROMs between the two work areas. This implies that there are more obstacles in the way of clinically implementing PROMs than mere knowledge and awareness. Our data analysis showed that the two main reasons for not using PROMs by orthopedic surgeons in this study were that PROM implementation needs significant changes to the structure of the clinical routine of healthcare providers and in turn leads to further costs (61.8%), and that there is no time space for regular implementation (63%).

Whereas orthopedic surgeons who collected PROMs from their patients were found to have similar motives, such as prioritizing clinical problems (97%) and facilitating communication between surgeons and patients (94%). This high level of agreeability suggests that once a successful implementation of PROMs has taken place, the focal point of treatment and care further shifts towards the patient, rather than the disease.

Age was associated with both knowledge and use of PROMs (*p* < 0.05), demonstrating that older orthopedic surgeons are more receptive towards PROMs. This is in contrast to a previous study by the AO Foundation that found no significant influence between the level of clinical experience and the use of PROMs, which further corroborate their speculation regarding collection of PROMs by less experienced clinicians on behalf of more senior ones in order to explain such a finding. Additionally, while PROM use by surgeons was found to be generally low in other countries, Saudi Arabian orthopedic surgeons are comparatively lower [14].

Surgical specialization may factor in both familiarity and use, especially in fields with well-established PROMs. Zwiers et al. (2018) reported that 188 feet and ankle surgeons were familiar with 20 different PROMs, and 72% of the respondents used PROMs, with 39% of them using it in regular clinical routine [17]. In contrast, Joeris et al. (2018) found that craniomaxillofacial surgeons had both low familiarity (31.7%) and use (15.4%), which was attributable to the lack of well-established PROMs for their patients [14]. In our study, Saudi orthopedic surgeons did not subscribe to this well-known link in the literature. While there are well-established PROMs for joint function and pain such as the Oxford Knee Score [18] and the Oxford Hip Score [19], orthopedic surgeons in Saudi Arabia remain relatively less familiar with these disease-specific PROMs (58%) with little use in clinical work (13.74%). This may imply that training programs, seminars, courses, and other forms of continuing medical education related to orthopedics in Saudi Arabia have not yet discussed PROMs sufficiently or at length. Interestingly, geographical regions have also been shown to affect familiarity and use, especially in countries that have started a PROM collection program, such as the Patient-Reported Outcomes Measurement Information System (PROMIS) program in the United States of America (USA) [20], and the national PROM program in the United Kingdom (UK) [21]. This may be a more likely explanation for the relatively poor rates of Saudi orthopedic surgeons’ familiarity and use of PROMs in comparison with their counterparts in North American and European countries since Saudi Arabia has yet to start a similar collection program.

One of the main limitations this study faces is the inability to cover MOH hospitals in other regions as well as private hospitals. While there is a notable effort to grant further regional autonomy on the directorate level, MOH hospitals remain largely uniform in policies and protocol [22]. Compounding the aforementioned with the fact that Riyadh and the Eastern Province are two of the largest regions in Saudi Arabia, it should be safe to assume that variation among other regions should not be statistically significant. In contrast, private hospitals are wholly independent, and there might be incentive to use PROMs due to contractual agreements with health insurance companies, as is the case in other countries [23].

Another limitation is the removal of two items in the branching logic part of the adapted questionnaire without revalidating the survey to ensure cultural compatibility, such as an item regarding the existence of regulations mandating PROM use in the surgeon’s hospital. The original survey’s target population was surgeons from multiple specialties such as spine, trauma, craniomaxillofacial, and orthopedics from countries all over the world. While the international nature of the original study gave little reason to revalidate the survey for local Saudi orthopedic surgeons, it is possible that future studies with a more culturally mindful design may yield new findings.

Further research in other regions to validate assumptions revealed by this study is needed, as well as examining in further detail the specific structure of clinical routine in MOH hospitals, which many orthopedic surgeons cited as requiring significant change for a successful implementation of PROMs.

## Conclusion

Our survey reveals that regular use of PROMs among orthopedic surgeons was exceptionally low, even though the majority were interested in using them. The main reasons included the lack of knowledge, the belief that collecting PROMs is too time-consuming, and that it requires a significant as well as costly overhaul to the structure of their clinical activity. One way to overcome these obstacles in MOH hospitals is to implement an integrated computer-based collection system and to further highlight the clinical importance of such tools and how to use them.

## Data Availability

The datasets used during the current study are available from the corresponding author on reasonable request.

## Abbreviations

PROMs: Patient-reported outcome measures
TJA: Total joint arthroplasty
MOH: Ministry of Health
